# Promoting safe walking among older people: the effects of a physical and cognitive training intervention vs. physical training alone on mobility and falls among older community-dwelling men and women (the PASSWORD study): design and methods of a randomized controlled trial

**DOI:** 10.1186/s12877-018-0906-0

**Published:** 2018-09-15

**Authors:** Sarianna Sipilä, Anna Tirkkonen, Tuomo Hänninen, Pia Laukkanen, Markku Alen, Roger A. Fielding, Miia Kivipelto, Katja Kokko, Jenni Kulmala, Taina Rantanen, Sanna E. Sihvonen, Elina Sillanpää, Anna Stigsdotter-Neely, Timo Törmäkangas

**Affiliations:** 1 Gerontology Research Center and Faculty of Sport and Health Sciences, University of Jyväskylä, Jyväskylä, Finland; 2 Department of Neurology, Kuopio University Hospital, Kuopio, Finland; 3 Department of Medical Rehabilitation, Oulu University Hospital, Oulu, Finland; 4 Nutrition, Exercise Physiology, and Sarcopenia Laboratory, Jean Mayer USDA Human Nutrition Research Center on Aging, Tufts University, Boston, MA USA; 5 Department of Public Health Solutions, Chronic Disease Prevention Unit, National Institute for Health and Welfare, Helsinki, Finland; 6 Division of Clinical Geriatrics, Center for Alzheimer Research, NVS, Karolinska Institutet, Stockholm, Sweden; 7 Institute of Clinical Medicine/Neurology, University of Eastern Finland, Kuopio, Finland; 8 Neuroepidemiology and Ageing Research Unit, School of Public Health, Imperial College London, London, UK; 9 School of Health and Social Studies, Jyväskylä University of Applied Sciences, Jyväskylä, Finland; 10 Department of Social and Psychological Studies, Karlstad University, Karlstad, Sweden; 11 Department of Psychology, Umeå University, Umeå, Sweden

**Keywords:** Aging, Executive function, Physical activity, Prevention, Sedentary

## Abstract

**Background:**

Safe and stable walking is a complex process involving the interaction of neuromuscular, sensory and cognitive functions. As physical and cognitive functions deteriorate with ageing, training of both functions may have more beneficial effects on walking and falls prevention than either alone. This article describes the study design, recruitment strategies and interventions of the PASSWORD study investigating whether a combination of physical and cognitive training (PTCT) has greater effects on walking speed, dual-task cost in walking speed, fall incidence and executive functions compared to physical training (PT) alone among 70–85-year-old community-dwelling sedentary or at most moderately physically active men and women.

**Methods:**

Community-dwelling sedentary or at most moderately physically active, men and women living in the city of Jyväskylä will be recruited and randomized into physical training (PT) and physical and cognitive training (PTCT). The 12-month interventions include supervised training sessions and home exercises. Both groups attend physical training intervention, which follows the current physical activity guidelines. The PTCT group performes also a web-based computer program targeting executive functions. Outcomes will be assessed at baseline and at 6 and 12 months thereafter. Falls data are collected during the interventions and the subsequent one-year follow-up. The primary outcome is 10-m walking speed. Secondary outcomes include 6-min walking distance, dual-task cost in walking speed, fall incidence and executive function assessed with color Stroop and Trail Making A and B tests. Explanatory outcomes include e.g. body composition and bone characteristics, physical performance, physical activity, life-space mobility, fall-related self-efficacy, emotional well-being and personality characteristics.

**Discussion:**

The study is designed to capture the additive and possible synergistic effects of physical and cognitive training. When completed, the study will provide new knowledge on the effects of physical and cognitive training on the prevention of walking limitations and rate of falls in older people. The expected results will be of value in informing strategies designed to promote safe walking among older people and may have a significant health and socio-economic impact.

**Trial registration:**

ISRCTN52388040.

## Background

A major task facing aging societies is to develop effective strategies to promote and improve older people’s functional capacity and engagement in society. Among the most important factors is the ability to walk safely and independently in one’s environment. Safe walking facilitates a physically and socially active life and access to goods and services [1].

Safe and stable walking is a complex process involving the interaction of neuromuscular, sensory and cognitive functions [2, 3]. The physiological prerequisites for walking are lower body muscle strength and power, postural balance and endurance [4]. Of the higher-order cognitive functions, better executive functions correlate with better walking ability and less falls among community-dwelling older people [2, 5, 6]. Executive functions control processes that support effective, flexible and goal directed behavior relying on a network of brain regions including prefrontal and parietal cortices and striatum [7, 8]. As physical and cognitive functions deteriorate with ageing, promoting these functions may help maintain safe walking among older people.

Physical activity is most likely a key factor in promoting safe walking in older people; however, the current scientific evidence is partially conflicting. A recently published large-scale trial showed that supervised moderate intensity training reduced disability risk, improved walking speed and physical performance but not cognitive functions among 70- to 89-year-old participants at risk for disability [9–11]. In another study, a resistance-training intervention resulted in significant improvements in muscle power but not in walking speed among 65- to 75-year-old women compared to a balance and toning control group [12]. Interestingly, in the latter study improvements in muscle power were accompanied by improvements in executive functions. Greater improvements in executive functions were also associated with better maintenance of physical activity over the one-year follow-up. Moreover, a falls prevention program including supervised strengthening, balance and functional exercises improved muscle strength and mobility in 70- to 80-year-old women with a history of falls, but had no effects on the overall rate of falls [13].

The interplay between higher cognitive functions and walking suggest that not only physical but also cognitive training has potential benefits for the prevention of mobility limitation and falls in older people. It may be that physical training and cognitive training induce additive or synergistic effects when combined in the same intervention. Physical training increases neurogenesis, angiogenesis and upregulates neurotrophic factors [14], while cognitive training increases recruitment of neurons and neuronal networks [15].

Research on the effects of cognitive training on walking among older people is scarce. Two pilot studies suggest that a computer-based program targeting executive functions may have effects on an untrained task with a large neuromuscular component [16, 17]. The precise underlying mechanisms remain unclear, but evidence from elsewhere shows that transfer may occur if the training and the transfer tasks share common processes and involve the same brain areas [18]. Earlier studies show that the frontal lobe, especially the dorsolateral prefrontal cortex is activated by both walking and executive functioning [2, 19] and that both cognitive and physical training increases dopamine release and hence may be one mechanism by which they interact [20].

This article describes the study design, recruitment protocol and interventions of a parallel group randomized controlled trial (RCT) among 70–85-year-old community-dwelling sedentary or moderately physically active men and women. The goal is to determine whether a combination of physical and cognitive training (PTCT) has greater effects on walking speed, dual-task cost in walking speed and executive functions compared to physical training (PT) alone. The effects of the PTCT intervention, compared to PT alone, on rate of falls during the 12-month intervention and subsequent one-year follow-up will also be investigated.

## Methods/design

### Study design

The PASSWORD study is a single site RCT with two research arms; Physical Training (PT, control) and a combination of Physical and Cognitive Training (PTCT) and conducted at the university laboratory. The interventions last for 12 months. Outcomes will be assessed at baseline and at 6 and 12 months thereafter. Falls data are collected during the 12-month interventions and during the subsequent one-year follow-up. Participants will be randomized into groups of equal size after the baseline assessments by a senior researcher who is not involved in the data collection or conducting the interventions of this study. A computer-generated random allocation sequence of two-fold stratification by gender and age (70–74, 75–79, 80–85) with randomly varying blocks of two and four will be utilized. Investigators collecting outcome data are blinded to the study group allocation. The participants are instructed not to talk about the group assignment with the personnel collecting the data. The study protocol has been designed according to CONSORT guidelines and it has been registered in the International Standard Randomized Controlled Trial Number Register (http://www.isrctn.com/ISRCTN52388040). Ethics approval for the study was received from the review board at the Ethical Committee of Central Finland Health Care District (14/12/2016, ref.: 11/2016). We give an information letter, explaining the details of the study, possible risks, and permission to use the data for research purposes, participants’ right to decline to participate at any point, anonymity and confidentially of the data to all potential participants. Before the baseline measurements and before signing the informed consent, each participant has opportunity to ask questions related to the study protocol from a trained research assistant, research nurse or the Principal Investigator.

### Participants and recruitment

Community-dwelling sedentary or at most moderately physically active, 70- to 85-year-old men and women living in the city of Jyväskylä, Finland will be recruited. The current level of physical activity is assessed by structured questions during the telephone interview. The acceptable level is less than 150 min per week of moderate physical activity and no regular resistance training during the past year. The inclusion and exclusion criteria are presented in Table 1.

**Table 1:** Inclusion and exclusion criteria of the PASSWORD study

Inclusion criteria	Exclusion criteria
Age 70 to 85	Severe chronic condition or medication affecting cognitive and/or physical function:
Community-dwelling	-cancer requiring treatment in the past year (except for basalioma, cancers that have been cured or carry an excellent prognosis)
Able to walk 500 m without assistance (cane is allowed)	-severe musculoskeletal (e.g. osteoarthritis, osteoporosis with fragility fracture) disease
	-severe lung, renal or cardio-vascular disease, diabetes with insulin medication
Sedentary or at most moderately physically active (less than 150 min of walking/week and no regular attendance in resistance training)	-severe psychotic disorder, cognitive impairment or disease affecting cognition (e.g. Alzheimer’s disease, dementia, abnormal CERAD score),
	-serious neurological disease or disorder (e.g. Parkinson’s disease), stroke or cerebral hemorrhage with complications
MMSE ≥24 Informed consent to participation	Underlying diseases likely to limit lifespan and/or intervention safety. Contraindication for physical exercise or walking tests based on ACSM^41^
	Other medical, psychiatric, or behavioral factor that in the judgment of the PI and study physician may interfere with study participation or the ability to follow the intervention protocol
	Excessive and regular use of alcohol (more than 7 units per week for women and 14 for men)
	Difficulty in communication due to severe vision or hearing problems
	Unable or unwilling to give informed consent or accept randomization into either study group
	Another member of the household is a participant in PASSWORD

*MMSE* Mini mental state examination test, *MCI* Mild cognitive impairment, *ACSM* American college of sports medicine

**Fig. 1:**
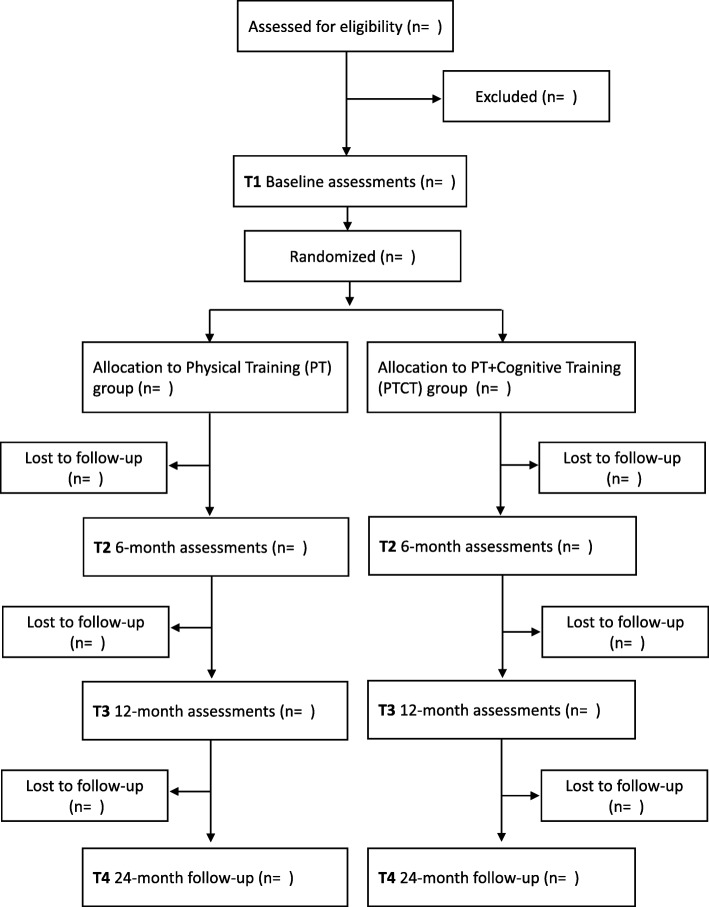
Flow chart of the PASSWORD study

Participants are randomly selected from the Finnish National Registry. Recruitment starts with a letter containing information about the study and an announcement to expect a phone call during the following week. Phone numbers are collected from a nationwide database. Repeat phone calls are made if necessary. The purpose of the phone interview, using standardized questions, is to screen for inclusion and exclusion criteria related to mobility, physical activity and major chronic diseases. In addition, the Short Nutritional Assessment Questionnaire (SNAQ) is used to assess the risk for malnutrition. Those who score two or more points in the SNAQ, will respond to the Mini Nutritional Assessment (MNA-SF) to ensure safe participation in the training intervention. Depression (Geriatric Depression Scale) and cognitive impairment (Mini Mental State Examination, MMSE, and Consortium to Establish a Registry for Alzheimer’s Disease, CERAD) will be assessed and health status confirmed by a nurse and, if necessary, a physician and clinical psychologist before the baseline assessments. Flow chart in shown in Fig. 1.

### Description of measurements

#### Primary outcome

The primary outcome is 10-m walking speed. Participants are asked to walk as fast as possible over the 10-m course. The time to complete the walk is measured by photocells. The test will be done twice and the best performance documented as the result. For the analysis, maximal walking speed (m/s) will be calculated. The test-retest precision with a 1- to 2-week interval in our laboratory is 5% [21]. Low walking speed is associated with e.g. increased risk for disability, cognitive impairment, institutionalization and falls [22].

#### Secondary outcomes

Secondary outcomes are 6-min walking distance, dual-task cost in walking speed, fall incidence and executive function. In *the 6-min walking test*, participants are encouraged to walk up and down a 20-m circuit for 6 min at a comfortable speed and without resting [23]. Rate of perceived exertion (RPE; [24]) will be assessed before the walk and at three and 6 min thereafter. The 6-min walking test serves as a measure for community walking and is associated with, e.g., mobility limitation and disability [25].

*The dual-task walking* test is adopted from that used by Menant et al. [26]. Participants are first asked to walk at a self-selected speed along a 20-m long walkway. They are then asked to repeat the walk while performing a visuospatial cognitive task. We measure walking times by photocells and then calculate the difference between the two walks (dual-task cost). The visuospatial task involves a display with three boxes side by side labelled A, B and C. Participants are asked to visualize a star located in one of the boxes making three movements. Prerecorded instructions deliver the random starting position and the direction of the three movements, i.e. left or right. The cognitive task instructions are delivered continuously throughout the walking trial through headphones. A new instruction will be delivered within 1 s of the participant answering the previous question. Participants practice the visuospatial task carefully before the dual-task walking test.

We monitor *fall incidence* by monthly diaries throughout the study. We define a fall as an unexpected event in which the participant comes to rest on the ground, floor or lower level without overwhelming extrinsic cause. For each fall, detailed information on its location and need for care due to the fall is reported. The participant will send the monthly calendar back to the study coordinator during the first week of the following month.

*Executive functions* are assessed by the color Stroop Test [27] and Trail Making A and B [28]. The Stroop Test is a test for inhibition and it includes three test conditions. First, participants are instructed to read out 72 words printed in black ink. Second, they are instructed to read out the color of 72 colored letter X’s. Finally, they are shown a page with 72 color words printed in incongruent colored inks (e.g., the word “RED” printed in blue ink). Participants are asked to name the color in which the words are printed and ignore the word itself. Participants are asked to do the test as quickly and as accurately as possible. The time taken to read each condition is recorded and the time difference between the third and the second condition calculated. Smaller time differences indicate better performance.

Trail Making Tests Parts A and B are used to assess set shifting. We instruct the participants to perform both parts as quickly and as correctly as possible. Part A assesses psychomotor speed. Participants are instructed to draw a line connecting circles with numbers 1 to 25 sequentially. Part B consists of circles with numbers and letters; participants are instructed to draw a line from 1 to A, A to 2, 2 to B, B to 3 etc., until they reach letter L. The time to complete each task is recorded and the time difference between Part B and A calculated. The smaller the difference, the better the performance.

#### Exploratory outcomes

*Overall health* including chronic diseases, medication, vision, blood pressure at resting and during orthostatic test, and resting EKG is assessed during a nurse’s examination. Information on chronic conditions and medication is collected by self-report and from the integrated patient information system utilized by the national health services (Effica database) by the study physician at baseline. If considered necessary, an examination by a study physician will be arranged after the nurse’s examination. Blood count, C reactive protein, and hemoglobin are measured to ascertain safe participation in the laboratory assessments and the intervention. Serum samples are stored for further analysis of inflammatory and growth factors.

*Anthropometrics and body composition* is measured by standard procedures and dual-energy x-ray absorptiometry (DXA, LUNAR Prodigy, GE Healthcare). Body height and weight are measured and a body mass index calculated. Total body composition and proximal femoral neck bone characteristics are measured by DXA. Subjects are scanned in supine position in the center of the table using the default-scanning mode for total body and proximal femoral region automatically selected by the Prodigy software (Lunar Prodigy Advance Encore v. 14.10.022).

*Perceived difficulty in walking* outdoors, 500 m and two kilometers is assessed with standard questions. The response options are: Able to manage without difficulty, Able to manage with some difficulty, Able to manage with a great deal of difficulty, Able to manage only with the help of another person, Unable to manage even with help [29].

*Life-space mobility* is assessed with the Finnish version of the University of Alabama at Birmingham Study of Aging Life-Space Assessment (LSA) [30, 31]. The LSA comprises 15 items and assesses mobility through the different life-space levels (bedroom, other rooms, outside home, neighborhood, town, beyond town) during the preceding 4 weeks. For each level, participants report how many days a week they attained that level and if they needed help from another person or assistive devices. Four indicators, with higher scores indicating a larger life-space, are calculated. 1) Independent life-space indicates the highest level of life-space attained without help from any devices or persons. 2) Assisted life-space indicates the highest level of life-space attained using the help of assistive devices if needed but not the help of another person. 3) Maximal life-space indicates the greatest distance attained with the help of devices and/or persons if needed. 4) A composite score reflects the distance, frequency and level of independence travelled (range 0–120).

*Physical performance* assessments include the Short Physical Performance Battery (SPPB), isometric knee extension and grip force and lower body extension power [32]. The SPPB includes habitual walking speed over four meters, five-time chair rise, and standing balance tests. The maximum score is 12, with higher scores indicating better performance. Those who score the maximum in the original SPPB balance test will repeat the test on a soft platform.

Maximal isometric knee extension force on the side on the dominant hand is measured using an adjustable dynamometer chair (Good Strength, Metitur Ltd., Palokka, Finland). The ankle is attached to a strain-gauge with the knee angle fixed at 60 degrees from full extension. The leg is extended as forcefully as possible and participants are encouraged to make a maximal effort during each trial [33]. Dominant handgrip force is measured with the dynamometer fixed to the arm of the chair with the elbow flexed at 90°. Participants are encouraged to squeeze the handle as hard as possible. Both force measurements are repeated three time or until no further improvement occurs. The highest force is used for analysis [33].

Leg extension muscle power is measured with the Nottingham Leg Extensor Power Rig from both legs [34]. Muscle power is a product of force and velocity, and it refers to the ability to produce force quickly. The measurement is repeated until no further improvement occurs and the best performance is used for analysis.

*Physical disability* is assessed by a validated questionnaire estimating perceived difficulties in six basic activities of daily living (ADL) which are eating, transferring from/to bed, dressing, bathing, cutting toe nails, and toileting [35, 36]. We also assess difficulties in eight instrumental activities of daily living (IADL), which are preparing meals, doing laundry, coping with light housework, coping with heavy housework, handling medication, using the telephone, using public transportation, and handling finances [36, 37]. Each ADL and IADL item includes five response categories: able to manage without difficulty, able to manage with some difficulty, able to manage with major difficulty, able to manage only with the help of another person, and unable to manage even with help.

*Fall-related self-efficacy* is assessed by the Falls Efficacy Scale International (FES-I; [38]). The questionnaire comprises 16 items assessing, e.g., walking on slippery, uneven or sloping surfaces, and visiting friends or relatives or going to a social event. Concern about falling when carrying out each activity is assessed on a four-point scale (range 1 = not at all concerned to 4 = very concerned). The total FES-I score ranges from 16 to 64. Fall-related self-efficacy describes perceived self-confidence in avoiding falls during every day activities [39].

*Neuropsychological tests* include global cognitive function as assessed by CERAD total score [40], and verbal fluency (Letter Verbal Fluency Test, [41]). The CERAD is composed of five subtests: Category Verbal Fluency, Modified Boston Naming Test (BNT), Mini Mental State Examination (MMSE), Word List Memory, and Constructional Praxis. The total score (range 0–100) is calculated according to the original procedure developed by Chandler et al. [42]. For the letter fluency task, participants are instructed to verbally generate as many words as possible that began with the letters P, A, and S in three separate 1-min trials.

*Psychological function* include tests for emotional well-being and personality characteristics. Emotional well-being is assessed using the Satisfaction with Life Scale [43] and Internationally Reliable Short Form of the Positive and Negative Affect Schedule (I-PANAS-SF, [44]). The Satisfaction with Life Scale consists of five items (e.g., “In most ways my life is close to my ideal”; response scale from 1 = strongly disagree to 7 = strongly agree), of which a sum score will be calculated. The I-PANAS-SF consists of ten adjectives (five for positive affectivity, e.g., “enthusiastic” and five for negative affectivity, e.g., “hostile”; response scale from 1 = does not describe my mood at all to 5 = describes me very well). Separate sum scores for positive and negative affectivity will be calculated. Personality traits are assessed using a short form of the Eysenck Personality Inventory modified by Floderus [45, 46] with 19 items with response scale 1 = no, 2 = yes (ten for extraversion, e.g., “Are you lively and talkative?” and nine for neuroticism, e.g. “Do you often feel apathetic and tired without any special reason?”). Separate sum scores for extraversion and neuroticism will be computed. The NEO-Personality Inventory-3 is also used (NEO-PI-3; [47]) for investigating personality traits. It has 240 items (response scale from 1 = strongly disagree to 5 = strongly agree), 48 for each personality trait (neuroticism, extraversion, openness to new experiences, conscientiousness, and agreeableness). Each of these five traits have six facets. Sum scores for each trait and their facets will be computed. Sense of coherence was measured using the 13-item version of Antonovsky’s [48] scale (e.g., “You anticipate that your personal life in the future will be…1 = totally without meaning or purpose to 7 = full of meaning and purpose”).A sum score of the items will be computed.

*The level of physical activity and sedentary behavior* is assessed by validated questions [49] and an accelerometer. Accelerometer recording is performed over seven consecutive days with a hip worn device (UKK, Tampere, Finland). The UKK device measures and stores acceleration in three orthogonal (x, y and z) directions at a sampling rate of 100 Hz. The accelerometers are returned by mail to the research institute where the stored data is copied to a hard disk for later analysis.

### Interventions

Interventions start with a 60–90-min introductory seminar, including a motivational lecture on the benefits of physical activity in older people. In addition, a description of the physical activity intervention and an individual time schedule for the supervised sessions are given. Participants have also an opportunity to ask questions and to communicate their expectations and possible challenges they face regarding participation in the study. The PTCT participants also attend the introductory seminar during which detailed information on cognitive training is given.

The interventions include supervised training sessions and home exercises. Supervised sessions are organized weekly in groups of 10–15 participants. Training adherence is carefully monitored and a daily diary recording all home exercises is kept. During the first 2 months, physical training only is organized. This arrangement facilitates participants’ adaptation to the training. The acceptability of the interventions is assessed by a questionnaire after the intervention. For the post-intervention follow-up, encouragement to continue physical and cognitive training, but no support, is given.

#### The multicomponent physical training (PT) intervention

PT intervention will be adapted from the physical activity guidelines for older adults, our earlier studies [33, 50] and the Lifestyle Interventions and Independence for Elders (LIFE) study [51]. The PT intervention will include aerobic physical exercises (mostly walking) and progressive resistance and balance training. Five to six different training periods with variation in training specificity, volume and intensity [52] are designed to maintain physiological responses to training and to prevent overtraining and fatigue during the 1-year training intervention. Detailed descriptions of the periods are shown in Table 2.

**Table 2:** Description of the multicomponent physical training intervention of the PASSWORD –study

Time, months	Programs/RM tests	Supervised resistance/balance exercise program	Supervised walking/balance exercise program	Home gymnastic program
1–2	6RM tests	Familiarization with equipment; RM for Leg press, Leg curl, Leg extension	150 min of aerobic exercise/week. Outdoors activities are encouraged throughout the intervention	
	Period 1 (adoption phase)	Warm-up with balance exercises; Resistance training at 50% of 1RM, 2 × 20 reps (adoption phase)	Warm-up (walk at habitualspeed and dynamic balance exercises while walking); 10-min continuous walk with RPE 13	Strength exercises for lower limb muscles; Postural balance exercise; Stretching exercises for major muscle groups
3–4	Period 2	Warm-up with balance exercises; *Resistance training*: resistance at 60% 1RM, 2 × 15 reps	Warm-up (at habitual speed, dynamic balance exercises of increasing difficulty over time while walking); 10–15 min continuous walking with RPE 13	Strength exercises for lower limb muscles; Postural balance exercise; Stretching exercises for major muscle groups
5–6	Period 3	Warm-up with balance exercises; *Power training:* Resistance 50% 1RM, 3 × 5 reps (fast contractions) *Hypertrophy:* Resistance 70% 1RM, 2 × 10 reps (resistance is increased by 1–2 kg if predefined number of reps is exceeded)	Warm-up (as in periods 3–4); 15–20-min continuous walk with RPE 13	Strength exercises for lower limb muscles with *red TheraBand CLX*; Postural balance exercise; Stretching exercises for majormuscle groups
	6RM tests	Leg press, Leg curl, Leg extension Agility training for two weeks	1 month break during summertime	
7–8	Period 4	Warm-up with balance exercises; *Hypertrophy:* Resistance training at 70% 1RM, 3 × 10 reps (resistance is increased by 1–2 kg if predefined number of reps is exceeded)	Warm-up (as in periods 3–4) 20-min continuous walk with RPE 13	Strength exercises for lower limb muscles with *green/blue Thera Band CLX*; Postural balance exercise; Stretching exercises for major muscle groups
9–10	Period 5	Warm-up with balance exercises; *Hypertrophy:* Resistance 80%, 1–2 × 10 reps (resistance is increased by 1–2 kg if predefined number of reps is exceeded) *Power:* Resistance 60%, 1–2 × 6–8 (fast contractions)	Warm-up (as in periods 3–4); 20-min continuous walk with RPE 13 or 20-min walk with < 1 min intervals with RPE 15	Strength exercises for lower limb muscles with *blue TheraBand CLX*;Postural balance exercise; Stretching exercises for major muscle groups
	6RM tests	Leg press, Leg curl, Leg extension		
11–12	Period 6	Warm-up with balance exercises; *Power:* Resistance 60%, 3 × 6 reps (fast contractions) *Hypertrophy:* Resistance 80%, 2 × 10 reps (resistance is increased by 1–2 kg if predefined number of reps is exceeded)	Warm-up (as in periods 3–4); 20-min walk with < 1 min intervals with RPE 15	Strength exercises for lower limb muscles with *blue TheraBand CLX*; Postural balance exercise; Stretching exercises for major muscle groups

Supervised walking sessions are organized once a week outdoors on a 400-m circular walking lane and, during the wintertime, indoors in a sports hall with a 200-m oval track. Throughout the study, walking sessions begin with a 10–15 min warm-up, including a short walk at self-selected speed and dynamic balance exercises of increasing difficulty to be performed while walking. After the warm-up, continuous walking for 10–20 min, at a target intensity of 13–15 (somewhat hard to hard) on the Borg scale [24], is performed.

Resistance training takes place in three senior gyms equipped with HUR senior line resistance training machines utilizing air pressure technology and Smart Card/Smart Touch Software (http://www.hur.fi/en). During the 12-month intervention, six different training periods, aimed at increasing muscle strength and power, are performed. Each training session includes 8–9 exercises for the lower body, trunk and upper body muscles. Leg press, leg curl and leg extension exercises form the core of the training program. Six-repetition maximum (6RM) tests for these exercises are performed during the first training session and after the 3rd and 5th training periods to determine training load. In addition, hip adduction and abduction, hip extension and heel rise as well as rowing, chest press or elbow extension are performed interchangeably during the training sessions. The training load for these exercises is self-selected by the participants with the aim of performing the same number of sets and repetitions as for the core exercises. Each resistance training session starts with a 10-min warm-up, including balance exercises, which increase in difficulty during the study.

The progressive home exercise program includes a structured gymnastic program with strengthening exercises for the lower limb muscles, postural balance exercise and stretching for major muscle groups. In the strengthening exercises, workload is increased with resistance bands of three different strengths. The standing balance exercises include heel and toe rise, semi- and tandem standing, standing on one leg, line walking and figure-of-eight walking. The level of challenge is increased by reducing hand, base and vision support. Participants are also advised to accumulate moderate aerobic activity amounting to a total of 150 min per week in bouts of at least 10 min duration. Recommended activities include walking, Nordic walking, biking and cross-country skiing.

#### Cognitive computer-based training (CT)

CT targets executive functions, namely inhibition, shifting and updating of working memory, and is built on the unity/diversity model of executive functions proposed by Miyake et al. [8]. The CT program is a web-based in-house developed computer program (iPASS) modified from that used in the Finnish Geriatric Intervention Study to Prevent Cognitive Impairment and Disability (the FINGER) study [18, 53]. The target training frequency is 3–4 times a week. CT starts with supervised group sessions organized in the University computer classroom and supervised by a student with, at least, psychology as a minor subject. During the first weeks of CT, peer support for the requisite computer skills is organized in collaboration with the GeroNet tutors of the local University of the Third Age. Participants who have the necessary computer skills and a computer at home, are allowed to start CT at home after 2–3 group sessions. Those who lack access to a computer at home can attend supervised sessions at least once a week and will also have the possibility to train in one of ten locations provided by the City of Jyväskylä (libraries, sheltered accommodation, etc.). In each location, a GeroNet tutor will be present each week for 2–3 h at a time.

During each training session, four different tasks are practiced. The tasks are organized in two blocks, which alternate between sessions. Task difficulty increases during the intervention period. Block 1 includes letter updating, predictable set-shifting, spatial working memory maintenance, and stroop color tasks (inhibition). Block 2 includes spatial updating, unpredictable set-shifting, spatial working memory maintenance, and stroop number tasks (inhibition). Participants are instructed to do the tasks as quickly and as accurately as possible. One training session lasts approximately 20 min.

#### Participant safety and data quality assurance

Participant safety is a priority in this study. The screening process ensures safe participation in the assessments and interventions. Supervisors and the personnel will be carefully trained for collecting the data and participants safety (including e.g. first aid course). Adverse events and falls will be tracked throughout the 12-month intervention, with special emphasis on events that could be associated with the study. Diseases, symptoms, and medication arising during the intervention are self-reported every 3 months. The study physician, study nurse and principal investigator will review these reports and make the decision on modification of the intervention and, if necessary, the decision to terminate the trial. All participants and personnel will be covered by insurance taken out by the University.

Our research center has a long tradition in administering physical activity interventions and mobility, physical and cognitive function measures among older populations. A Standard Operation Procedure document will be carefully followed throughout the study. Periodical meetings of the study group and checks will be set up to monitor data collection quality. During participants’ laboratory visits, all questionnaires are reviewed by the staff. Where information is missing, participants are asked to complete the questionnaire. Information collected on paper will be saved as data files as soon as possible. Each participant will be assigned an identification number. The identification -key will be in the possession of the research coordinator, research nurse and principal investigator (PI) during the data collection and thereafter in the possession of the PI only. All collected data will be stored on the University server and protected by passwords. Data collection form are available at https://www.jyu.fi/sport/fi/tutkimus/hankkeet/password

### Statistical analysis and sample size

The effects of the intervention will be assessed on the intention-to-treat principle. Maximum likelihood methodology will be used to account for missing data. The primary outcome will be tested for group-interaction over time using an interaction contrast in a linear model for longitudinal data accounting for within-person correlation and different variances at the two time-points. Negative binomial regression will be used to estimate the incident rate ratio for falls. The Cox proportional hazard regression model will be used to calculate hazard rates up to the first fall for fallers in both groups with PT as a reference. In addition, individual changes in the main and secondary outcomes observed during the study will be calculated and a reliable change index computed. The effects of the intervention on primary and secondary outcomes will also be evaluated in sub-analyses stratified by age, gender, baseline cognition, and level of compliance to the intervention.

A priori sample size calculations were based on previously published data [13, 16, 17] and on our own data on 10-m walking speed. We expect a baseline mean level of 1.3 m/s (standard deviation of 0.36 m/s) in both groups [53, 54]. The PT intervention is expected to induce a four-percentage point mean increase in both groups and a six-percentage point higher mean in the PTCT group than PT group with no change in standard deviation (SD). The follow-up within-person correlation for the two measurements is estimated to be *r* = 0.80 [49], yielding 0.23 m/s as an estimate of the SD for change. Setting the significance level at 0.05 and power at 80% for the group-time interaction favoring the PTCT vs. the PT group indicated that a sample size of 135 participants per group is required. Given an anticipated dropout level of 15%, we decided to recruit 155 participants per group. An additional power analysis, based on recently published data [13] was calculated for the secondary outcome of the falls rate. At 80% power and with a total sample size of 270–310, it would be possible to detect a difference of 27–29% in falls rate significant (α = 0.05) between the PT and PTCT groups.

### Current status

As of April 1st 2018, 314 participants have been recruited and randomized to study groups.

## Discussion

The purpose of the PASSWORD study, conducted among 70–85-year-old community-dwelling sedentary or at most moderately physically active men and women, is to investigate whether physical and cognitive training combined has greater effects on walking speed, dual task cost in walking speed, executive function and fall incidence than physical training alone. The study is designed to capture the additive and possible synergistic effects of physical and cognitive training by using evidence-based guidelines, training regimens, and a comprehensive battery of validated tests. When completed, the study will provide new knowledge on the additive effects of physical and cognitive training on the prevention of walking limitations and rate of falls in older people. The expected results will be of value in informing strategies designed to promote safe walking among older people. The results may also have a significant health and socio-economic impact.

The current scientific evidence on the effects of physical training on safe walking and falls prevention is encouraging but partially conflicting. Some have shown benefits for walking but not for cognition [9–11], while others have yielded the opposite results [12]. Moreover, mixed results have been reported on the role of physical activity on fall incidence [13]. Observational data indicate, that cognitive training has potential for the prevention of mobility limitation and falls in older people [2, 6]. Data on experimental designs is, however, scarce. A few earlier studies have investigated the effects of physical and cognitive training interventions on walking speed or the rate of falls compared with physical activity alone [55]. However, the training regimens used did not follow any existing guidelines or evidence-based regimens and the studies themselves were small-scale.

The PASSWORD study utilizes interventions which rely on evidence-based findings and which have been tested in large scale trials with good compliance and results [9, 53]. Supervised sessions aim at maximal training effects whereas home exercises facilitate adaptation to an active lifestyle. The PASSWORD physical training intervention is based on current guidelines for older adults. To benefit health and well-being across a broad spectrum, muscle strengthening and balance training as well as moderate intensity aerobic activities are performed regularly on a weekly basis.

The cognitive training program is a modification of that already used in a large multicenter study, the FINGER study [53]. Multidomain cognitive training activates larger neuronal networks and is more likely to elicit transfer effects than single domain training. It also enables variation in training sessions, which may increase training compliance. Moreover, computer-based exercises enable individually adjusted progression by increasing the level of difficulty over time.

In long-term trials, participant commitment to the study protocol may be challenging and may change over the trial. Travelling to organized, supervised sessions on a weekly basis over a lengthy trial may, for several reasons, most typically diseases, lack of time and loss of interest, be too demanding for many older people. Hinrichs et al. [56] found that in a physical activity intervention study of only 12 weeks duration involving community-dwelling mobility-limited older people, 47% of the participants reported 151 adverse events. Only two of the events were related to the intervention. This indicates that although a training program has been carefully designed and is safe, high morbidity unrelated to the intervention can constitute a critical challenge for sustained participation.

To foster adherence to the planned interventions and measurements, we carefully review participants’ health and cognition, fully inform them about the procedures and interventions and give them opportunities to ask questions concerning the study before randomization. Moreover, we add the different components of the intervention gradually, over a period of 6–8 weeks, to the training program. This gives the participants time to adapt to the intervention routines. Regular supervised group sessions may help to engage participants in the group activity and thus in the training protocol. As not all participants are expected to have computer skills or a computer at home, peer support and possibility to train out of home is provided throughout the study.

In conclusion, the evidence on the effects of physical training interventions on walking speed, falls prevention and cognition among community-dwelling at most moderately physically active older people is contradictory. Therefore, research investigating new strategies to promote safe walking in older populations is needed. The PASSWORD study is a randomized controlled trial designed to capture the synergistic effects of a combination of physical and cognitive training on safe walking compared to physical training alone. The physical training intervention chosen for the control condition follows the current guidelines (“standard care”) for older adults. A unique feature of PASSWORD is that it uses proven interventions in a novel combination and a robust set of mobility, falls and executive function measurements. The results of the PASSWORD study are expected to influence guidelines on the prevention and treatment of mobility limitations and disabilities among older people and thus inform future health care practices and policies.

## Abbreviations

ADL: Activities of daily living
CERAD: Consortium to Establish a Registry for Alzheimer’s Disease
CT: Cognitive training
DXA: Dual-energy x-ray absorptiometry
EKG: Electrocardiography
FES-I: The Falls Efficacy Scale International
GDS: Geriatric Depression Scale
IADL: Instrumental activities of daily living
LSA: Life-Space Assessment
MMSE: Mini-Mental State Examination
MNA-SF: Mini Nutritional Assessment- Short Form
NEO-PI: NEO Personality Inventory-3
PT: Physical training
PTCT: Combination of physical training and cognitive training
RCT: Randomized controlled trial
Reps: Repetitions
RM: Repetition maximum
RPE: Rate of perceived exertion
SD: Standart deviation
SNAQ: Short Nutritional Assessment Questionnaire
SPPB: Short Physical Performance Battery
the FINGER-study: The Finnish Geriatric Intervention Study to Prevent Cognitive Impairment and Disability
the LIFE-study: The Lifestyle Interventions and Independence for Elders
